# Assessing the reproducibility of high temporal and spatial resolution dynamic contrast-enhanced magnetic resonance imaging in patients with gliomas

**DOI:** 10.1038/s41598-021-02450-5

**Published:** 2021-12-01

**Authors:** Woo Hyeon Lim, Joon Sik Park, Jaeseok Park, Seung Hong Choi

**Affiliations:** 1 Department of Radiology, Seoul National University College of Medicine, 28, Yongon-dong, Chongno-gu, Seoul, 110-744 Korea; 2 Department of Biomedical Engineering, Sungkyunkwan University, Suwon, Korea; 3 Department of Intelligent Precision Healthcare Convergence, Sungkyunkwan University, Suwon, Korea; 4 Center for Nanoparticle Research, Institute for Basic Science (IBS), Seoul National University, Seoul, 151-742 Korea; 5 School of Chemical and Biological Engineering, Seoul National University, Seoul, Korea

**Keywords:** CNS cancer, Cancer imaging

## Abstract

Temporal and spatial resolution of dynamic contrast-enhanced MR imaging (DCE-MRI) is critical to reproducibility, and the reproducibility of high-resolution (HR) DCE-MRI was evaluated. Thirty consecutive patients suspected to have brain tumors were prospectively enrolled with written informed consent. All patients underwent both HR-DCE (voxel size, 1.1 × 1.1 × 1.1 mm^3^; scan interval, 1.6 s) and conventional DCE (C-DCE; voxel size, 1.25 × 1.25 × 3.0 mm^3^; scan interval, 4.0 s) MRI. Regions of interests (ROIs) for enhancing lesions were segmented twice in each patient with glioblastoma (*n* = 7) to calculate DCE parameters (K^trans^, V_p_, and V_e_). Intraclass correlation coefficients (ICCs) of DCE parameters were obtained. In patients with gliomas (*n* = 25), arterial input functions (AIFs) and DCE parameters derived from T2 hyperintense lesions were obtained, and DCE parameters were compared according to WHO grades. ICCs of HR-DCE parameters were good to excellent (0.84–0.95), and ICCs of C-DCE parameters were moderate to excellent (0.66–0.96). Maximal signal intensity and wash-in slope of AIFs from HR-DCE MRI were significantly greater than those from C-DCE MRI (31.85 vs. 7.09 and 2.14 vs. 0.63; *p* < 0.001). Both 95^th^ percentile K^trans^ and V_e_ from HR-DCE and C-DCE MRI could differentiate grade 4 from grade 2 and 3 gliomas (*p* < 0.05). In conclusion, HR-DCE parameters generally showed better reproducibility than C-DCE parameters, and HR-DCE MRI provided better quality of AIFs.

## Introduction

Dynamic contrast-enhanced (DCE) magnetic resonance (MR) imaging is a well-validated MR imaging technique due to its usefulness in neuroimaging research. For example, several studies have suggested that DCE MR imaging-derived parameters (DCE parameters) have potential to differentiate the grades of gliomas^1–6^ and predict genetic mutation status^7–9^ or pseudoprogression after standard treatments^10,11^. In addition, DCE MR imaging has a potential role in other brain diseases, such as acute ischemic stroke^12^, multiple sclerosis^13^, dementia^14^, and traumatic brain injury^15^.

However, there are some limitations to apply DCE parameters directly to real clinical practice because of their low reproducibility^16^, primarily arising from the reproducibility of arterial input function (AIF) used in DCE MR imaging analysis^17–20^. Previously, You et al^21^ reported that the accuracy and reproducibility of DCE MR imaging parameters could be improved by using AIFs derived from dynamic susceptibility contrast (DSC) MR imaging. This improvement might be achieved by obtaining AIF with accelerated scan intervals, but gadolinium-based contrast agents (GBCAs) need to be injected twice to use DSC MR imaging-based AIF on DCE MR imaging analysis^21^.

Recently, Park et al^22^ introduced high resolution DCE (HR-DCE) MR imaging, in which z-axis resolution and temporal resolution were markedly improved compared to conventional DCE (C-DCE) MR imaging. This refinement in resolution of DCE MR imaging might improve the reproducibility of this imaging technique.

To the best of our knowledge, no previous study has evaluated HR-DCE MR imaging in glioma patients. Thus, this study aimed to evaluate the reproducibility and validate the usage of HR-DCE MR imaging in patients with gliomas compared with C-DCE MR imaging.

## Results

Table 1 shows demographics and pathologic results of study population. Examples of histograms and parametric maps of K^trans^ and V_e_ are displayed in Supplementary Fig. 1.

**Table 1:** Demographics and pathologic results of study population.

	Primary study (*n* = 7)	AIF analysis (*n* = 25)	Extended study (*n* = 15)
Sex	M : F = 4 : 3	M : F = 14 : 11	M : F = 9 : 6
Age (years)	54.3 ± 17.4	50.5 ± 14.4	47.9 ± 14.8
Pathologic diagnosis	Glioblastoma = 7	Glioblastoma = 13	Glioblastoma = 6
		Gliosarcoma = 2	Gliosarcoma = 2
		Diffuse midline glioma = 1	Anaplastic astrocytoma = 3
		Anaplastic astrocytoma = 5	Diffuse astrocytoma = 1
		Diffuse astrocytoma = 1	Oligodendroglioma = 3
		Oligodendroglioma = 3	
Ki-67 (%)	55.3 ± 16.0	36.5 ± 27.0	30.7 ± 28.1
MGMT promoter methylation	3	15	11
IDH1 mutation	0	8	7

*M* male, *F* female, *MGMT* O^6^-methylguanine-DNA-methyltransferase, *IDH* isocitrate dehydrogenase.

### Primary study

DCE parameters using population-based AIF showed excellent intraclass correlation coefficients (ICCs) of both C-DCE and HR-DCE MR imaging (range, 0.96–0.99) (Supplementary Fig. 2). When individual AIFs were used, ICCs of HR-DCE parameters showed good to excellent agreement (range, 0.84–0.95), while C-DCE parameters showed moderate to excellent agreement (range, 0.66–0.96) (Table 2).

**Table 2:** ICCs of DCE parameters derived from C-DCE and HR-DCE MRI in patients with glioblastoma (*n* = 7) using individual AIFs.

DCE parameter	1st C-DCE MRI 2nd C-DCE MRI	1st HR-DCE MRI 2nd HR-DCE MRI	Overall C-DCE MRI	Overall HR-DCE MRI
Mean K^trans^	0.71 (0.00, 0.94)* 0.66 (− 0.09, 0.93)	0.84 (0.33, 0.97) 0.93 (0.63, 0.99)	0.71 (0.36, 0.93)	0.92 (0.76, 0.98)
Mean V_p_	0.94 (0.70, 0.99) 0.95 (0.75, 0.99)	0.92 (0.62, 0.99) 0.91 (0.58, 0.99)	0.95 (0.85, 0.99)	0.94 (0.83, 0.99)
Mean V_e_	0.96 (0.80, 0.99) 0.70 (− 0.01, 0.94)	0.95 (0.73, 0.99) 0.93 (0.65, 0.99)	0.77 (0.46, 0.95)	0.95 (0.86, 0.99)

*ICC* intraclass correlation coefficient.

*ICC with 95th percentile confidence interval.

Correlations between C-DCE and HR-DCE parameters were not statistically significant (*p* > 0.05) in patients with glioblastoma (GBM). Both C-DCE and HR-DCE parameters were not statistically associated with pathologic results, such as Ki-67 values and O^6^-methylguanine-DNA-methyltransferase (MGMT) promoter methylation status (*p* > 0.05), in patients with GBM (Supplementary Table 1).

### AIF analyses

The 95th percentile of bolus arrival time (BAT) in C-DCE MR imaging was 20 s and that of wash-in time was 16 s. Thus, delayed bolus arrival error was defined when BAT was longer than 40 s (*n* = 1). In addition, delayed washout error was defined when time from maximal signal intensity (MSI) to half of MSI in wash-out slope (T_wash-out_) was longer than 32 s (*n* = 7).

**Figure 1:**
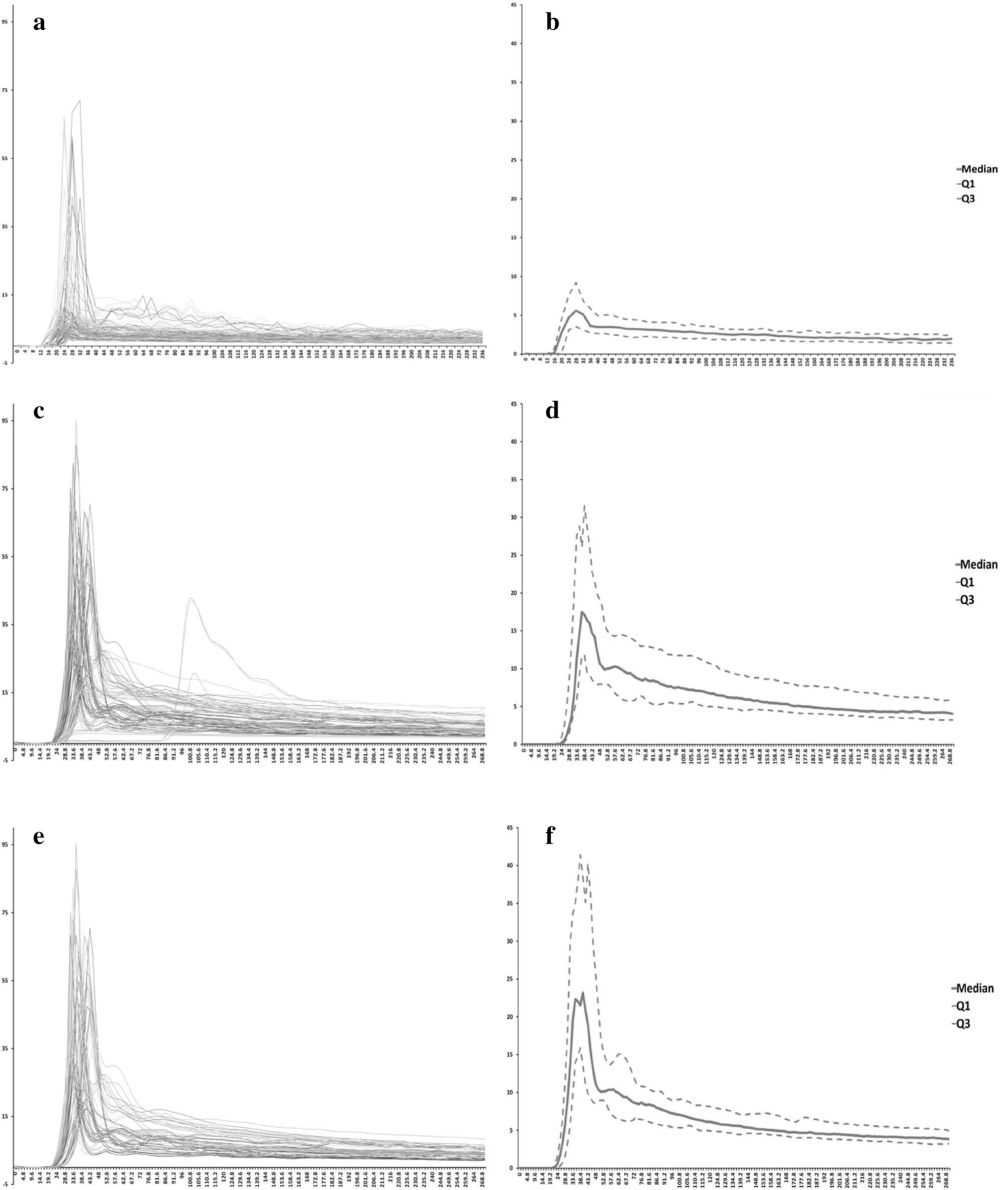
AIFs from C-DCE and HR-DCE MR imaging: (**a**) Individual AIFs of C-DCE MR imaging, (**b**) virtual AIFs derived from C-DCE MR imaging using median, Q1 and Q3 values, (**c**) individual and (**d**) virtual AIFs derived from HR-DCE MR imaging, (**e**) individual and (**f**) virtual AIFs derived from HR-DCE MR imaging after exclusion of suboptimal cases.

The MSI and wash-in slope (WIS) of AIFs from HR-DCE MR imaging were significantly greater than those of AIFs from C-DCE MR imaging (median, 31.85 vs. 7.09 and 2.14 vs. 0.63, respectively; *p* < 0.001) (Table 3), even when suboptimal AIFs were included. After exclusion of suboptimal AIFs, HR-DCE MR imaging still demonstrated greater MSI and steeper WIS of AIFs than C-DCE MR imaging (44.60 vs. 5.87 and 3.01 vs. 0.57, respectively; *p* < 0.001) (Table 3). All AIFs from 25 patients are plotted in Fig. 1.

**Table 3:** Comparison of AIF parameters derived from C-DCE and HR-DCE MRI.

	C-DCE MRI	HR-DCE MRI	*p*-value
**25 patients with 100 AIFs**			
BAT (s)	16.0 [12.0, 20.0]*	22.4 [20.8, 24.0]	N/A
TTP (s)	24.0 [24.0, 28.0]	38.4 [35.2, 41.6]	N/A
BSI	0.0 [− 0.03, 0.04]	0.0 [0.0, 0.0]	N/A
MSI	7.09 [5.34, 11.93]	31.85 [17.25, 53.74]	*p* < 0.001
WIS	0.63 [0.46, 1.30]	2.14 [1.04, 3.32]	*p* < 0.001
**Exclusion of suboptimal AIFs (*n* = 68)**			
BAT (s)	16.0 [12.0, 18.0]	20.8 [20.8, 24.0]	N/A
TTP (s)	24.0 [24.0, 28.0]	36.8 [33.6, 41.6]	N/A
BSI	0.0 [− 0.06, 0.01]	0.0 [0.0, 0.0]	N/A
MSI	5.87 [5.03, 10.75]	44.60 [29.39, 65.19]	*p* < 0.001
WIS	0.57 [0.44, 1.34]	3.01 [2.12, 3.99]	*p* < 0.001

*BAT* bolus arrivial time, *TTP* time to peak, *BSI* baseline signal intensity, *MSI* maximal signal intensity, *WIS* wash-in slope, *N/A* not applicable.

*Median[Interquartile range].

### Extended study

**Figure 2:**
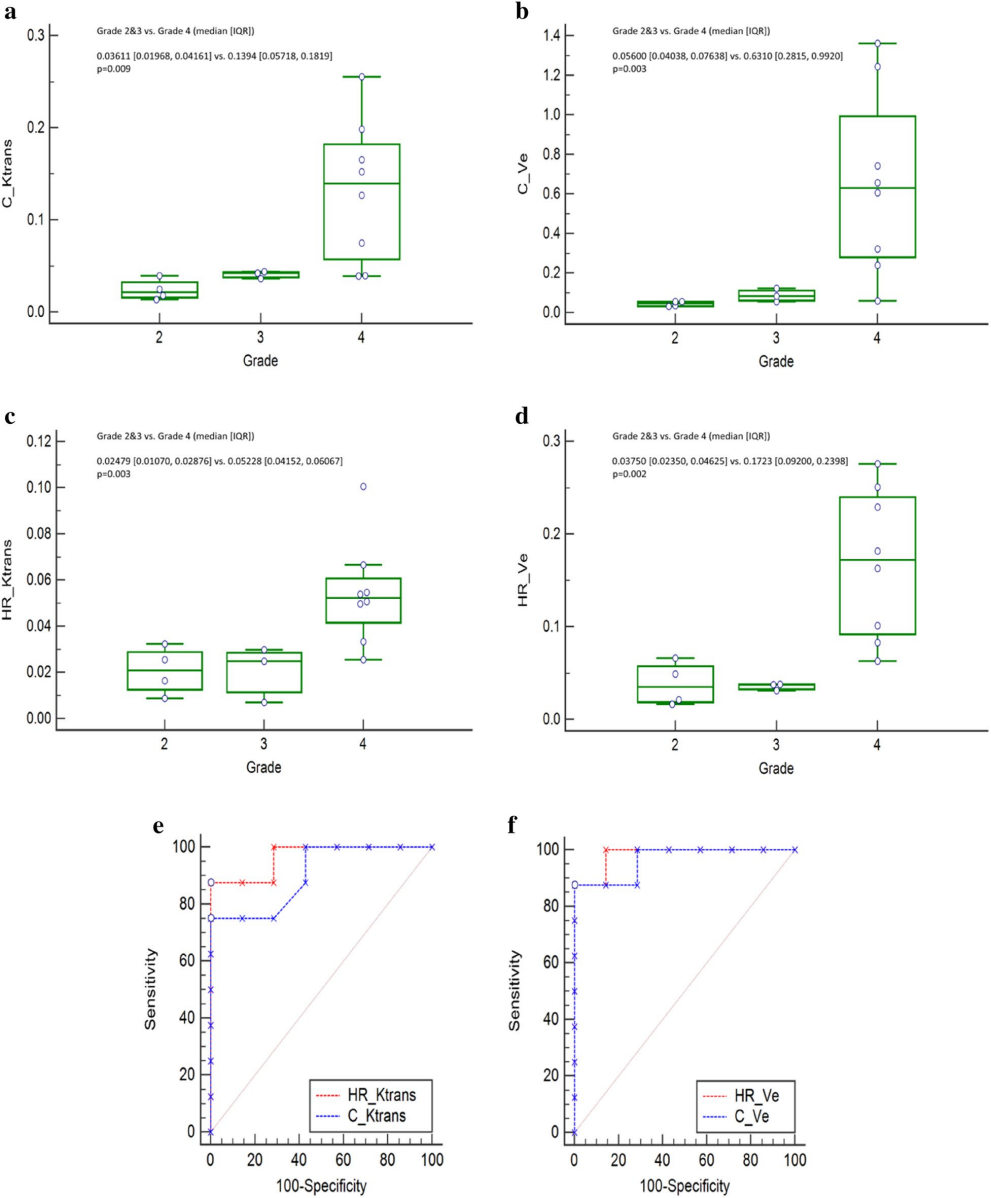
DCE MR parameters as differentiators of WHO tumor grades. (**a**) 95th percentile K^trans^ and (**b**) 95th percentile V_e_ according to tumor grades (grade 4 vs. grade 2 and 3) using C-DCE MR imaging, (**c**) 95th percentile K^trans^ and (**d**) 95th percentile V_e_ according to tumor grades (grade 4 vs. grade 2 and 3) using HR-DCE MR imaging, (**e**) ROC curves derived from K^trans^ and (**f**) ROC curves derived from V_e_ for differentiators of tumor grades.

ICCs of C-DCE and HR-DCE MR imaging were described in Supplementary Table 2. The 95th percentile K^trans^ and V_e_ from C-DCE imaging were correlated with those from HR-DCE MR imaging in patients with gliomas (Supplementary Fig. 3). Both 95th percentile K^trans^ and 95th percentile V_e_ derived from HR-DCE and C-DCE MR imaging could differentiate grade 4 gliomas from grade 2 and 3 gliomas (*p* < 0.05) (Fig. 2). Areas under the receiver operating characteristic curves (AUROC) were not significantly different (95th percentile K^trans^, C-DCE = 0.902 vs. HR-DCE = 0.964; *p* = 0.539; 95^th^ percentile V_e_, C-DCE = 0.964 vs. HR-DCE = 0.982; *p* = 0.738) (Fig. 2).

## Discussion

In this prospective study, we evaluated the reproducibility and clinical applicability of HR-DCE MR imaging in glioma patients, which demonstrated superior spatial (especially z-axis) and temporal resolutions compared to C-DCE MR imaging^22^. As many previous studies mentioned, acquisition of reliable AIF is a key factor in DCE MR imaging^16–21^, and improvement in the temporal resolution of DCE MR imaging^21^ could be a possible strategy to overcome the low reproducibility of this technique.

Several previous studies highlighted the importance of DCE MR imaging with higher temporal resolution in the prostate or breast^23–25^. Indeed, accelerated temporal resolution of DCE MR imaging in our study demonstrated the improved reproducibility of DCE parameters and superior ability to detect MSI of AIF in patients with gliomas. The effect of higher spatial resolution on the reproducibility of DCE parameters seems to be less significant.

Unexpectedly, HR-DCE MR imaging-specific errors (delayed wash-out error, delayed bolus arrival error) were noted. Even after thorough review and discussion with developers of HR-DCE MR imaging, we could not determine the exact cause of those errors. We believe there might be some setting errors during MR imaging that were not identified retrospectively. Nonetheless, HR-DCE MR imaging demonstrated the superior ability to detect greater MSI and steeper WIS of AIF. According to the study by You et al^21^, DSC MR imaging-based AIF showed improvement in the reproducibility of DCE MR imaging. Although this improvement was achieved by demonstrating higher signal intensity changes caused by the T2^*^ effect^21^, it is also plausible that accelerated scan interval (1.6 s vs. 4.0 s) enables us to catch the MSI of AIF more precisely.

Perfusion MRI has a role in treatment response evaluation in patients who underwent antiangiogenic agents (i.e., bevacizumab)^26–28^, but DCE parameters are not routinely used in real clinical practice because of their low reproducibility. Because the reproducibility of imaging parameters is a critical issue in clinical practice and trials^17,29,30^, our results of improved reproducibility of HR-DCE MR imaging could increase the possibility of implementing this technique in real clinical practice.

Interestingly, it seemed that histogram of DCE parameters could be different when temporal and spatial resolutions had been changed. Differences in z-axis resolution as well as AIF might affect the shape of histogram. Further evaluation investigating the effect of spatial and temporal resolutions on histograms is needed because histogram analysis has added prognostic value in patients with GBM^31^.

According to the results for spatial resolution, ICCs were excellent in both C-DCE and HR-DCE MR imaging, even though the z-axis resolution was significantly different. This might suggest that increased spatial resolution had a less significant effect on the reproducibility of DCE parameters. Indeed, HR-DCE MR images comprise 33,984 DICOM files in each patient, and heavy data size of HR-DCE MR imaging requires excessive analysis time. Although high spatial resolution of DCE MR imaging has an advantage in superior image quality^32^, clinical usefulness of DCE MR imaging might be enhanced with lower spatial resolution, given our results showing a less significant effect of spatial resolution on the reproducibility of DCE parameters.

In terms of clinical usefulness, the 95^th^ percentile K^trans^ and V_e_ were significantly higher in grade 4 gliomas than in grade 2 and 3 gliomas. However, the superiority of HR-DCE MR imaging for tumor grading was not proven because AUCs were not different in C-DCE and HR-DCE MR imaging. These findings might be related to the small size of the study population.

There are some limitations to our study. First, our study population was rather small because of several suboptimal cases, and this might be why our study failed to prove correlation between HR-DCE parameters and pathologic results in patients with GBM. Second, we could not identify the exact cause resulting in suboptimal AIFs. This finding might urge validation of HR-DCE MR imaging in different study populations. Third, our study did not evaluate whether HR-DCE parameters could predict survival better or differentiate pseudoprogression from true progression after treatment more precisely. Thus, our study has little clinical impact, but we focused on the clinical applicability of HR-DCE MR imaging in terms of reproducibility. Further studies pursuing the clinical importance of HR-DCE MR imaging in a larger population is needed. Finally, it is difficult to set a gold standard in DCE MR imaging studies. Thus, we chose C-DCE parameters as reference standards, and HR-DCE and C-DCE parameters did not always correlate with each other. Thus, DCE parameters should be interpreted with clinical context, which often failed to be demonstrated in our study, possibly due to the small study population. This also suggests the importance of clinical validation of HR-DCE MR imaging in a larger study population.

In conclusion, HR-DCE parameters showed better reproducibility than C-DCE parameters, and AIF derived from HR-DCE MR imaging exhibited higher MSI and steeper WIS in prospectively enrolled patients with gliomas. Further studies investigating clinical importance using HR-DCE MR imaging should be conducted with a larger study population.

## Materials and Methods

This prospective study was approved by the institutional review board of Seoul National University Hospital, and written informed consent was obtained from all patients, and all experiments were performed in accordance with relevant guidelines and regulations.

### Patient selection

From October 2018 to March 2019, 30 consecutive patients with presumed primary brain tumors who underwent diagnostic MR imaging were included in our study, and the details of diagnostic MR imaging protocol were previously described by You et al^21^. During diagnostic MR imaging, C-DCE MR images were routinely obtained. In these patients with informed consent, HR-DCE MR images were additionally obtained during navigation MR imaging for surgery. Because HR-DCE MR imaging was performed with GBCAs that used for navigation MR imaging, additional administration of GBCAs did not need. Median of time interval between diagnostic and navigation MR imaging was 2 days (interquartile range, 1–3 days).

**Figure 3:**
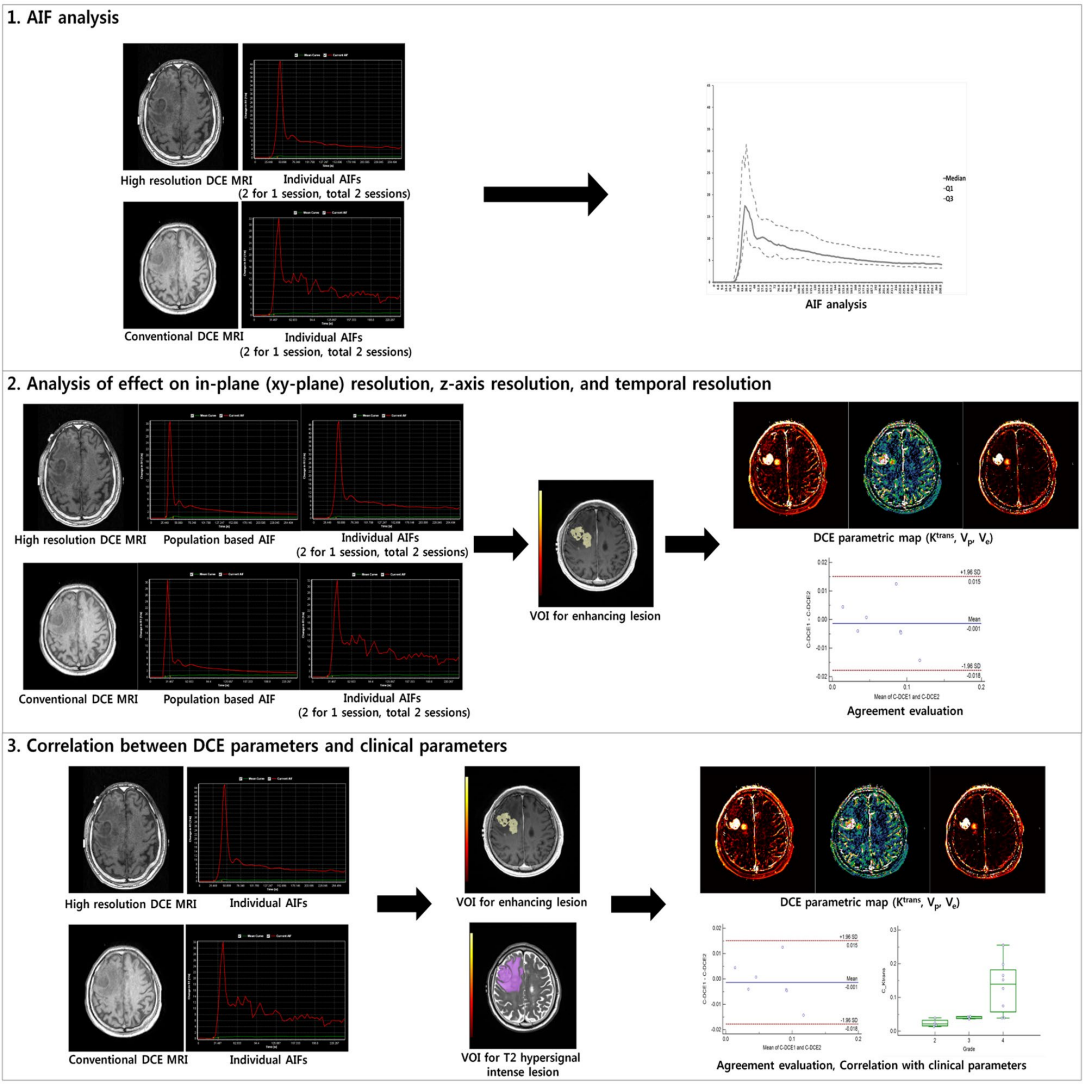
Study flow diagram. This study consists of (1) AIF analysis using individual AIFs, (2) analysis of the effect on in-plane (xy-plane) spatial resolution using population-based AIF, and the effect on the z-axis and temporal resolution using individual AIFs in patients with GBM, and (3) correlation between DCE parameters and clinical parameters using individual AIFs and VOIs for T2 hypersignal intense lesions.

The study design was abbreviated on Fig. 3 and Supplementary Fig. 4. The final pathological diagnosis was confirmed based on the WHO 2016 classification. For the primary study, we included patients whose final pathologic diagnosis was GBM (*n* = 14) and one patient was finally excluded whose HR-DCE MR imaging could not be analyzed. Thus, HR-DCE MR imaging data from 13 patients were included in the primary study. Among them, 6 patients were additionally excluded from the primary analysis, because of HR-DCE specific suboptimal AIFs (Supplementary Fig. 5). Other pathologies in 16 patients were as follows: gliosarcomas = 2, anaplastic astrocytomas = 5, diffuse astrocytomas = 2, diffuse midline glioma = 1, oligodendrogliomas = 3, metastasis = 1, lymphocytic infiltration = 1, and lymphoma = 1. For an extended study, we also included patients with other types of gliomas.

### MR imaging parameters

Glioma study MR imaging studies were performed by using 3.0 T MR imaging units (Verio or Skyra, Siemens Healthineers, Erlangen, Germany; Ingenia, Philips Heathcare, Best, Netherlands) with a 32-channel head coil, and HR-DCE MR imaging was performed by using either Verio or Skyra (Siemens Healthineers, Erlangen, Germany).

Glioma study MR imaging protocols included pre- and postcontrast three dimensional magnetization-prepared rapid acquisition with gradient echo (MPRAGE) sequences (Repetition time[TR]/Echo time[TE] = 1670/2.8 ms, Flip angle[FA] = 9^°^, Matrix = 256 × 232, Field of view[FOV] = 226 × 250, Section thickness = 1.0 mm, Number of excitation[NEX] = 1.0 for Siemens MR imaging machines; TR/TE = 8.1–8.2/3.7 ms, FA = 8^°^, Matrix = 240 × 240, FOV = 240 × 240, Section thickness = 1.0 mm, NEX = 1.0 for Philips MR imaging machine), transverse T2-weighted image (T2WI) with turbo spin echo (TSE) sequence (TR/TE = 2000–4630/80–299 ms, FA = 90–150^°^, Matrix = 252 × 252 or 512 × 464 or 640 × 297, FOV = 185 × 220 to 250 × 250, Section thickness = 4.0–5.0 mm, NEX = 1.0–2.0). Navigation MR imaging protocols consisted of postcontrast axial T1-weighted image (T1WI) with gradient echo (TR/TE = 1600/2.3 ms, FA = 9^°^, Matrix = 256 × 256, FOV = 240 × 240, Section thickness = 2.0 mm, NEX = 1.0), axial T2WI with TSE (TR/TE = 5700–6160/93.0 ms, FA = 130^°^, Matrix = 256 × 256, FOV = 240 × 240, Section thickness = 2.0 mm, NEX = 1.0), and HR-DCE MR imaging.

DCE MR imaging was performed by intravenous administration of gadobutrol (Gadovist, Bayer Schering Pharma, Berlin, Germany) at a dose of 0.1 mmol/kg of body weight. The specific imaging parameters for C-DCE MR imaging were as follows: TR/TE = 2.8/1.0 ms, FA = 10^°^, Matrix = 192 × 192, FOV = 240 × 240, Section thickness = 3.0, Voxel size = 1.25 × 1.25 × 3 mm^3^, NEX = 1.0, Scan interval = 4.0 s, Total images = 40 × 60 phases for Verio and Skyra scanners; and TR/TE = 4.2/2.1 ms, FA = 10^°^, Matrix = 192 × 192, FOV = 240 × 240, Section thickness = 3.0, Voxel size = 1.25 × 1.25 × 3.0 mm^3^, NEX = 1.0, Scan interval = 4.0 s, Total images = 40 × 60 phases for Ingenia Scanner. The specific imaging parameters for HR-DCE MR imaging were as follows: TR/TE = 3.2/1.4 ms, FA = 15^°^, matrix = 192 × 144, FOV = 229 × 172, section thickness = 1.1, voxel size = 1.1 × 1.1 × 1.1 mm^3^, NEX = 1.0, scan interval = 1.6 s, and total images = 192 × 177 phases.

### Image analysis for primary study

All image analyses were performed using commercially available software, Nordic ICE (NordicNeuroLab, Bergen, Norway), by a single radiologist (5 years of experience in neuroradiology) under the supervision of an expert neuroradiologist (18 years of experience in neuroradiology).

For C-DCE MR image analyses, parametric maps of K^trans^, V_p_, and V_e_ were generated based on extended Tofts model^33^, using C-DCE MR image digital imaging and communications in medicine (DICOM) files. AIF search box was drawn at the level of the middle cerebral artery transverse segment^34^, and AIF was generated as a mean of five different AIFs from 5 automatically selected pixels. The second AIF was generated by using an AIF search box to draw subsequent axial images in the same manner to evaluate the reproducibility of AIF. After coregistration between DCE parametric maps and postcontrast T1WI images^21,34^, regions of interest (ROI) were drawn to cover contrast-enhancing portion and avoid cystic portions or vascular structures for each transverse image. Using these ROIs with enhancing foci, volumetric information about DCE parameters was calculated on a pixel-by-pixel basis. HR-DCE MR image analyses were performed in the same manner using postcontrast T1WI from navigation MR imaging and HR-DCE MR image DICOM files.

After a month, AIF selections in two consecutive image planes were repeated to evaluate intraobserver reproducibility. Previously designated ROIs for enhancing lesions were reused to exclude the effect of ROI selection and assess interobserver reproducibility primarily arising from z-axis and temporal resolutions. DCE parametric maps and AIFs obtained from these measurements were used to evaluate the intraobserver reproducibility and AIF analysis.

In addition, to evaluate the reproducibility originating from spatial resolution, DCE parametric maps using population-based AIF were generated to reduce the effect of AIF selection, and a second ROI for measurable enhancing foci was drawn for each patient. Using two individual ROIs and DCE parametric maps derived from population-based AIF, intraobserver reproducibility related to in-plane resolution was evaluated.

### AIF analysis

As in a previous study^21^, five parameters from AIF were evaluated: (a) BAT, (b) time to peak (TTP), (c) baseline signal intensity (BSI), (d) MSI, and (e) WIS. AIF analyses were performed both with and without suboptimal AIFs. Virtual AIFs from the median value were plotted with 25th (Q1) and 75th percentile (Q3) values^21^.

In cases in which AIFs were significantly different from others, AIFs were only used for AIF analysis, while DCE parameters derived from those AIFs were not included for DCE parameter comparison and correlation with clinical parameters. In our study, two types of suboptimal AIFs were detected in HR-DCE MR imaging. To identify these findings, T_wash-out_ was calculated to define delayed wash-out error: T_wash-out_ > two times more than 95^th^ percentile value of wash-in time (time from BAT to TTP) using C-DCE MR images (Supplementary Fig. 5). Similarly, delayed bolus arrival error was defined when BAT was significantly delayed (two times more than the 95^th^ percentile value of BAT using C-DCE MR images) (Supplementary Fig. 5).

### Extended study

For extended study, T2WI images instead of postcontrast T1WI images were used as structural images. Using T2WI images, T2 hypersignal intense areas and contrast-enhancing lesions on postcontrast T1WI were included for ROI designation. DCE parametric map generation and coregistration were performed in the same manner as previously described. DCE parameters as differentiators for tumor grading were evaluated ^21^ in these patients. When there was a technical problem in coregistration of DCE and structural images, DCE parameter comparison or correlation with clinical parameters were not performed (Supplementary Fig. 4).

### Statistical analysis

ICC and Bland–Altman plotting were performed to evaluate the intraobserver reproducibility of DCE MR imaging according to spatial and temporal resolution. For ICC interpretation, ICC values were considered (a) poor (ICC < 0.50), (b) moderate (ICC, 0.50–0.75), (c) good (ICC, 0.75–0.90), and (d) excellent (ICC > 0.90)^35^. Linear correlation coefficient was evaluated between mean pharmacokinetic parameters derived from C-DCE MR imaging and those derived from HR-DCE MR imaging. Relationship between DCE parameters and pathologic results such as Ki-67 or MGMT promoter methylation status were compared with linear and logistic regression analyses.

Normality of the parameters was assessed using Kolmogorov–Smirnov test, and subsequently, comparisons of AIFs or DCE parameters were performed by paired *t* test or Wilcoxon test, as appropriate. Unpaired parametric and nonparametric data were compared by independent samples *t* test or Mann–Whitney test, as appropriate. Comparison of area of receiver operating characteristic curve (AUROC) was performed between grade 4 tumors and others.

The statistical analysis was performed using statistical software (MedCalc version 15.2, Ostend, Belgium), and *p*-values less than 0.05 were considered statistically significant.

## Supplementary Information

Supplementary Information 1.

Supplementary Information 2.

## Data Availability

Data of our study population could be accessed as a separate excel file.
