# Supplementary material for: Assessing the reproducibility of high temporal and spatial resolution dynamic contrast-enhanced magnetic resonance imaging in patients with gliomas
Source: Sci Rep. 2021 Dec 1;11:23217. doi: 10.1038/s41598-021-02450-5 (PMC8636480; doi:10.1038/s41598-021-02450-5)

Assessing the reproducibility of high temporal and spatial resolution dynamic contrast-enhanced magnetic resonance imaging in patients with gliomas

Woo Hyeon Lim[1)], Joon Sik Park[2), 3)], Jaeseok Park[2), 3)], Seung Hong Choi[1), 4), 5)*]

1) Department of Radiology, Seoul National University College of Medicine, Seoul, Korea

2) Department of Biomedical Engineering, Sungkyunkwan University, Suwon, Korea

3) Department of Intelligent Precision Healthcare Convergence, Sungkyunkwan University, Suwon, Korea

4) Center for Nanoparticle Research, Institute for Basic Science (IBS), Seoul National University, Seoul, Korea

5) School of Chemical and Biological Engineering, Seoul National University, Seoul, Korea

* Corresponding author: Seung Hong Choi

Supplementary Materials

Supplementary Table 1. Correlation of DCE parameters in patients with glioblastoma (n=7) using individual AIFs.

**C-DCE vs. HR-DCE**			
	**C-DCE**	**HR-DCE**	**P-value**
K^{trans}	0.05430 [0.03777, 0.09893]*	0.03239 [0.02413, 0.04235]	p=0.851
V_p	0.05497 [0.02846, 0.06732]	0.006936 [0.005984, 0.01089]	p=0.914
V_e	0.4538 [0.1362, 0.5411]	0.1191 [0.05613, 0.1964]	p=0.750

**Ki-67**		
	**C-DCE**	**HR-DCE**
K^{trans}	R^2=0.276, p=0.226	R^2=0.135, p=0.418
V_p	R^2=0.091, p=0.511	R^2=0.118, p=0.451
V_e	R^2=0.001, p=0.942	R^2=0.180, p=0.342

**MGMT promotor methylation**		
	**C-DCE**	**HR-DCE**
K^{trans}	p=0.311	p=0.213
V_p	p=0.469	p=0.310
V_e	p=0.701	p=0.445

Note> C-DCE=conventional dynamic contrast-enhanced MR imaging parameters, HR-DCE=high-resolution DCE parameters, MGMT=O^6-methylguanine-DNA-methyltransferase

* Median with interquartile range

Supplementary Table 2. ICCs of DCE parameters derived from C-DCE and HR-DCE MRI in patients with glioma (n=25) using individual AIFs.

DCE parameter	C-DCE MRI	HR-DCE MRI	C-DCE MRI according to grades	HR-DCE MRI according to grades
**Mean K^{trans}**	0.90 (0.73, 0.97)*	0.96 (0.88, 0.99)	Gr 4: 0.83 (0.37, 0.96)<br>Gr 3: -0.46 (-0.98, 0.87)<br>Gr 2: 0.91 (0.16, 0.99)	Gr 4: 0.93 (0.71, 0.99)<br>Gr 3: 0.95 (0.04, 1.00)<br>Gr 2: 0.78 (-0.31, 0.98)
**Mean V_p**	0.68 (0.27, 0.88)	0.90 (0.74, 0.97)	Gr 4: 0.75 (0.16, 0.94)<br>Gr 3: 0.98 (0.49, 1.00)<br>Gr 2: 0.63 (-0.56, 0.97)	Gr 4: 0.93 (0.69, 0.99)<br>Gr 3: 0.90 (-0.35, 1.00)<br>Gr 2: 0.84 (-0.13, 0.99)
**Mean V_e**	0.98 (0.94, 0.99)	0.95 (0.86, 0.98)	Gr 4: 0.97 (0.84, 0.99)<br>Gr 3: 0.92 (-0.24, 1.00)<br>Gr 2: 0.95 (0.47, 1.00)	Gr 4: 0.92 (0.67, 0.98)<br>Gr 3: 0.20 (-0.93, 0.97)<br>Gr 2: 0.89 (0.05, 0.99)

Note> ICC=intraclass correlation coefficient, Gr=grade

* ICC with 95th percentile confidence interval

Supplementary Figure 1. Examples of histograms and parametric maps of DCE MR imaging. Histograms and parametric maps from C-DCE MRI (a. histogram of K^{trans}, b. histogram of V_e, c. K^{trans} map, and d. V_e map) and Histograms and parametric maps from HR-DCE MRI (e. histogram of K^{trans}, f. histogram of V_e, g. K^{trans} map, and h. V_e map).

a)
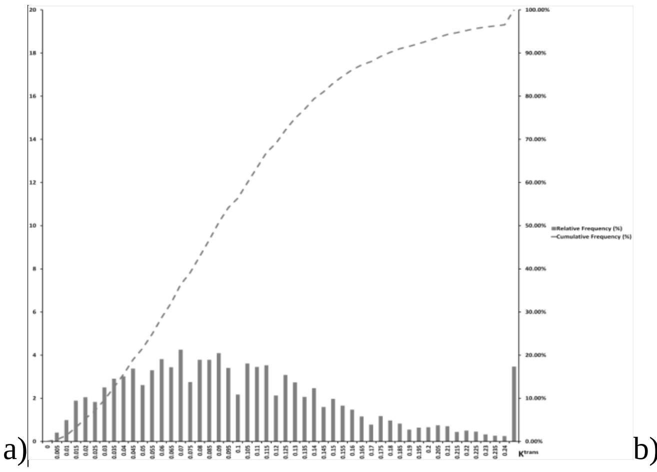

b)
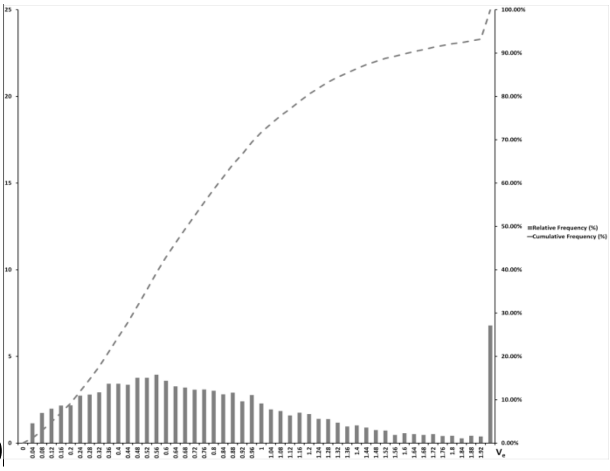

c)
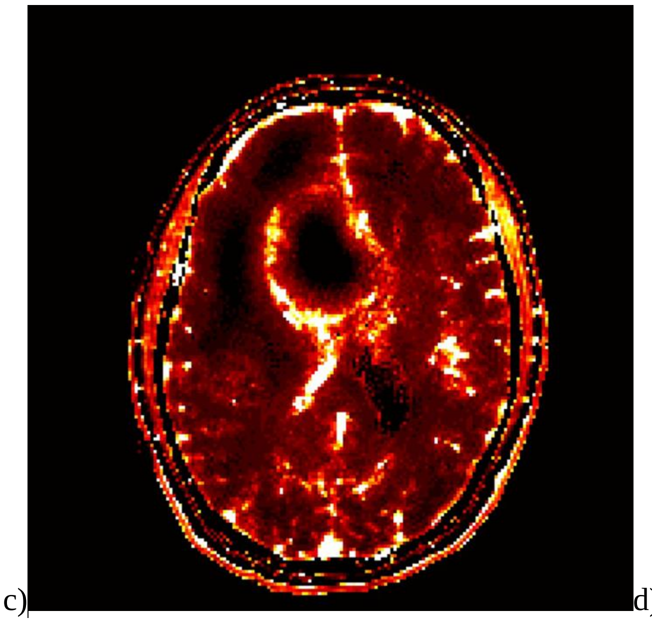

d)
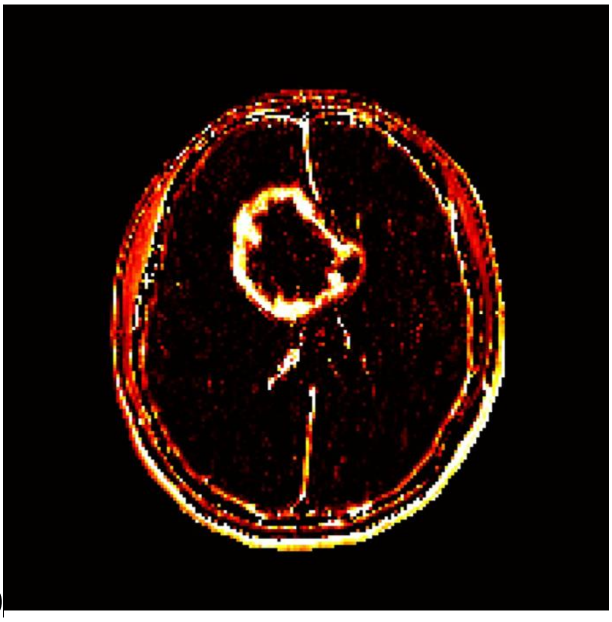

e)

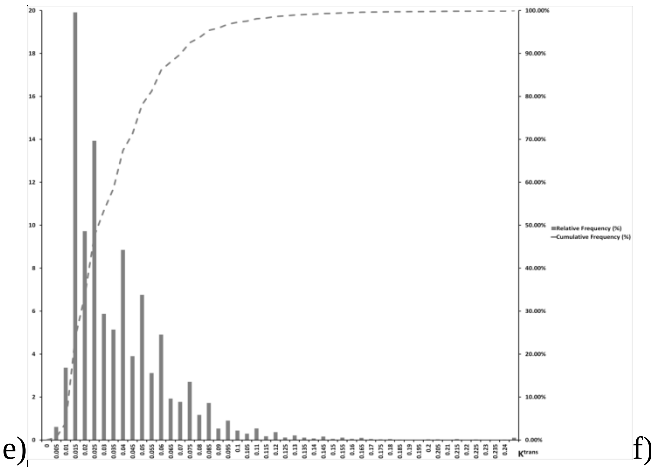
Relative Frequency (%)
Cumulative Frequency (%)
Ktrans

f)

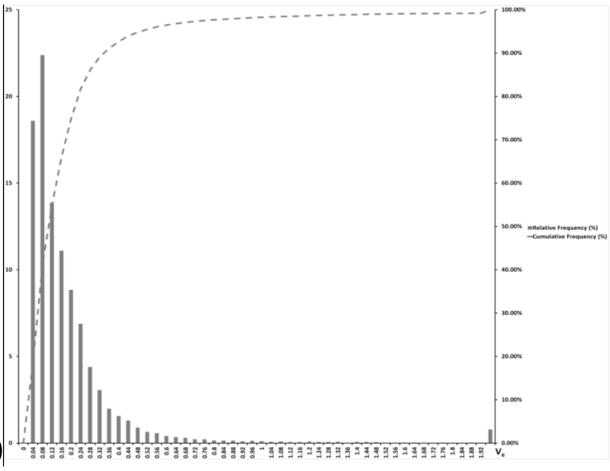
Relative Frequency (%)
Cumulative Frequency (%)
Ve

g)

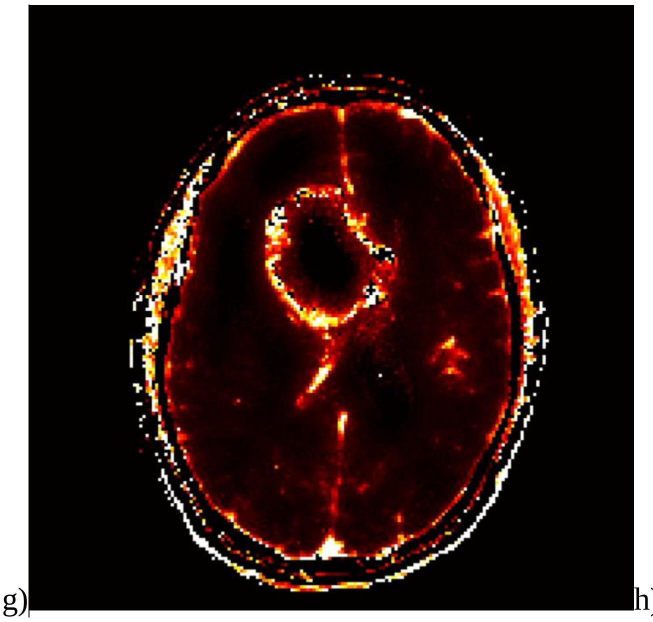

h)

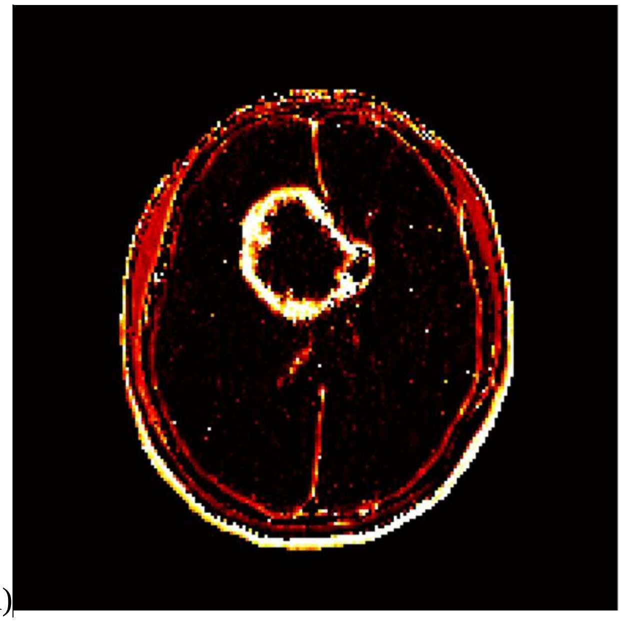

Supplementary Figure 2. Bland-Altman plots of DCE parameters: a) mean K^{trans} derived from C-DCE MRI (ICC=0.98), b) mean K^{trans} derived from HR-DCE MRI (ICC=0.98), c) mean V_p derived from C-DCE MRI (ICC=0.97), d) mean V_p derived from HR-DCE MRI (ICC=0.99), e) mean V_e derived from C-DCE MRI (ICC=0.98), and f) mean V_e derived from HR-DCE MRI (ICC=0.96) using population-based AIF in patients with glioblastoma.

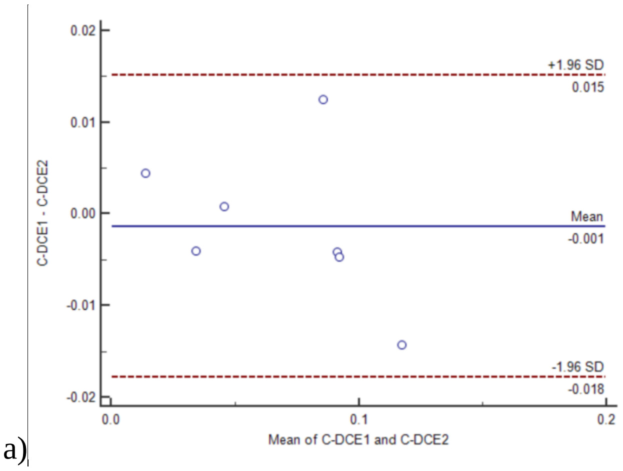

a)

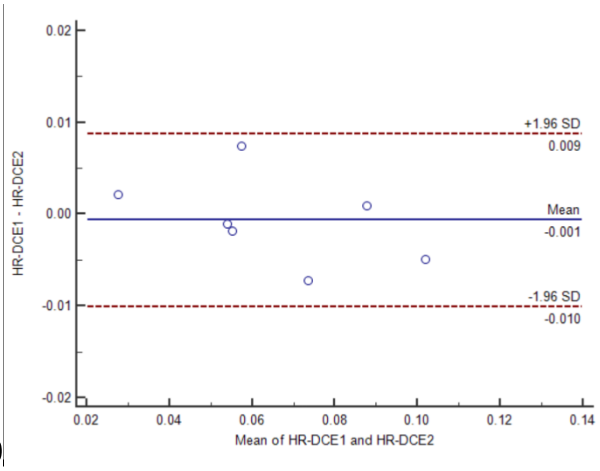

b)

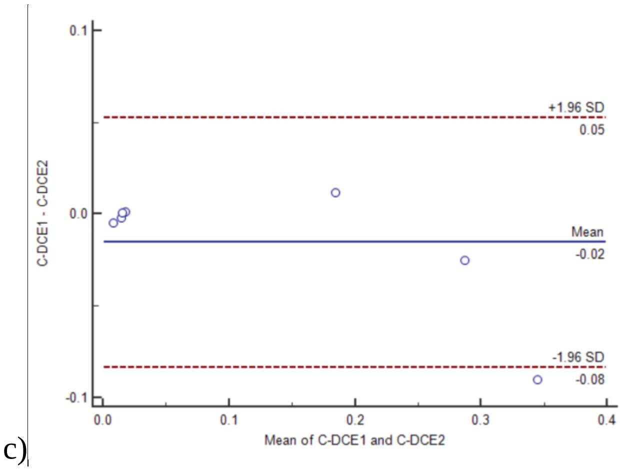

c)

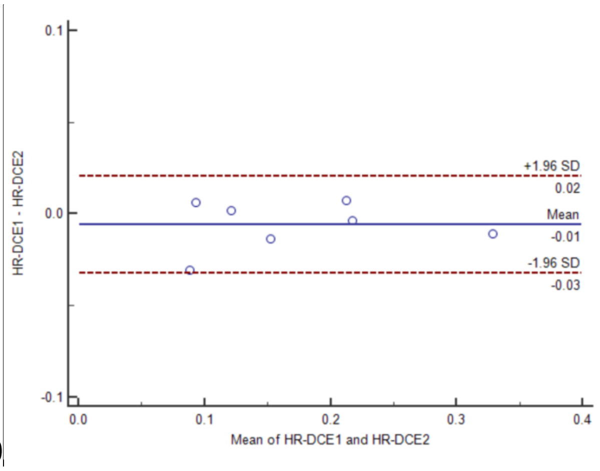

d)

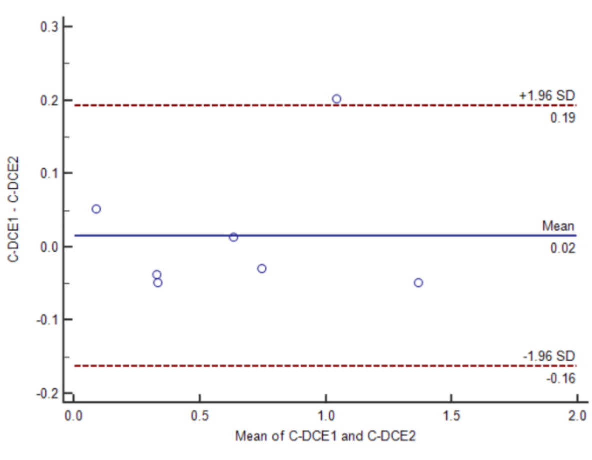
C-DCE1 - C-DCE2
Mean of C-DCE1 and C-DCE2
+1.96 SD
0.19
Mean
0.02
-1.96 SD
-0.16
0.3
0.2
0.1
0.0
-0.1
-0.2
0.0
0.5
1.0
1.5
2.0
e)

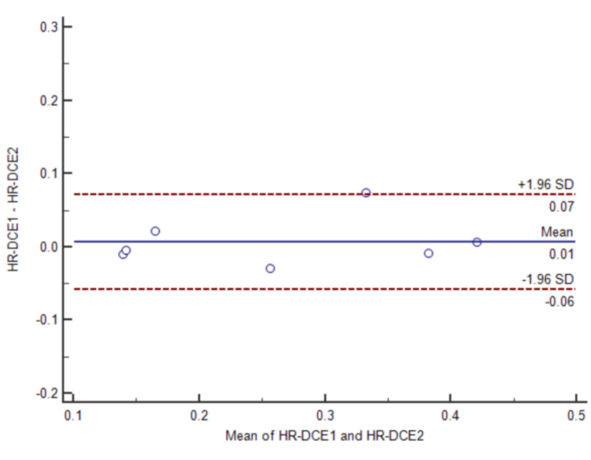
HR-DCE1 - HR-DCE2
Mean of HR-DCE1 and HR-DCE2
+1.96 SD
0.07
Mean
0.01
-1.96 SD
-0.06
0.3
0.2
0.1
0.0
-0.1
-0.2
0.1
0.2
0.3
0.4
0.5
f)

Supplementary Figure 3. Correlation between a) 95th percentile K^{trans} (R^2=0.284, p=0.041), b) 95th percentile V_p (R^2=0.004, p=0.820), and c) 95th percentile V_e (R^2=0.524, p=0.002) derived from C-DCE and HR-DCE MRI using VOIs for T2 hypersignal intense lesions.

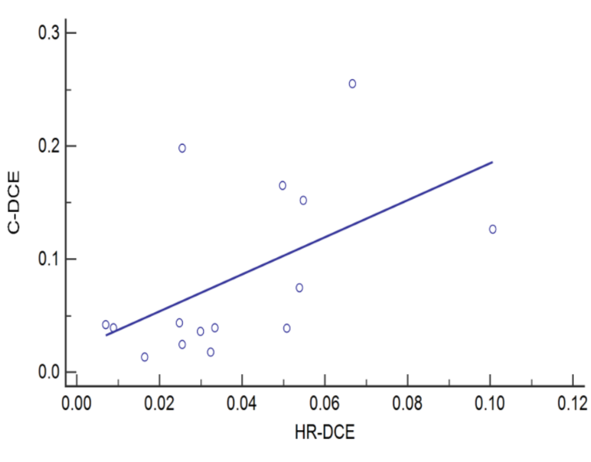

a)

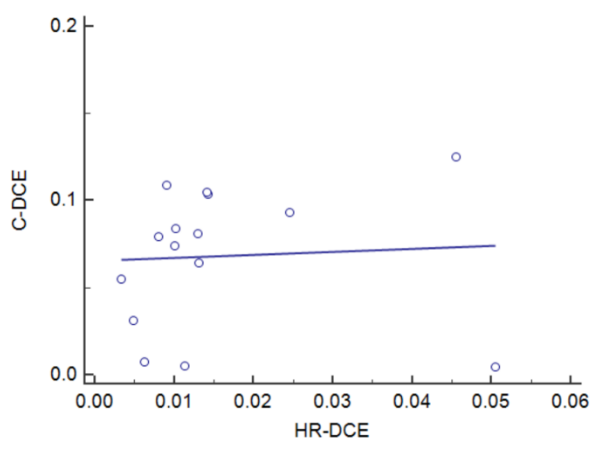

b)

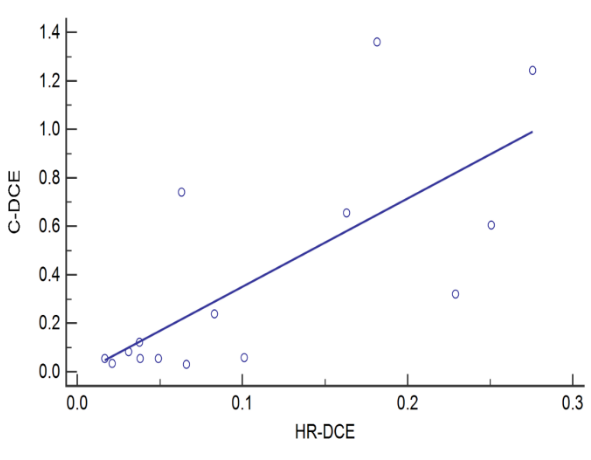

c)

Supplementary Figure 4. Study flow diagram.

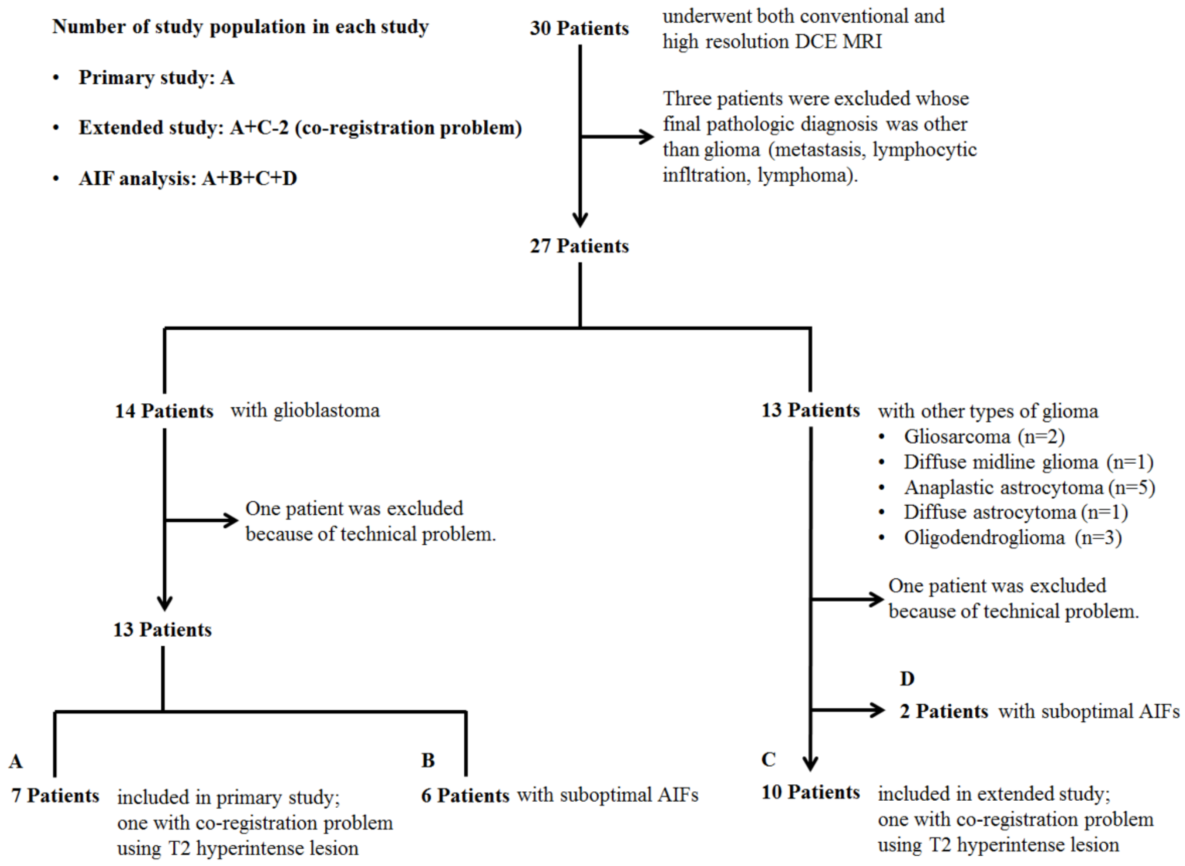

Supplementary Figure 5. Example of suboptimal cases in defection of AIF (delayed wash-out error): a) DCE map (5 red dots: measured points), b) calculated AIF, c) individual AIFs at 5 different points, d) all AIFs showing delayed wash-out or delayed bolus arrival errors.

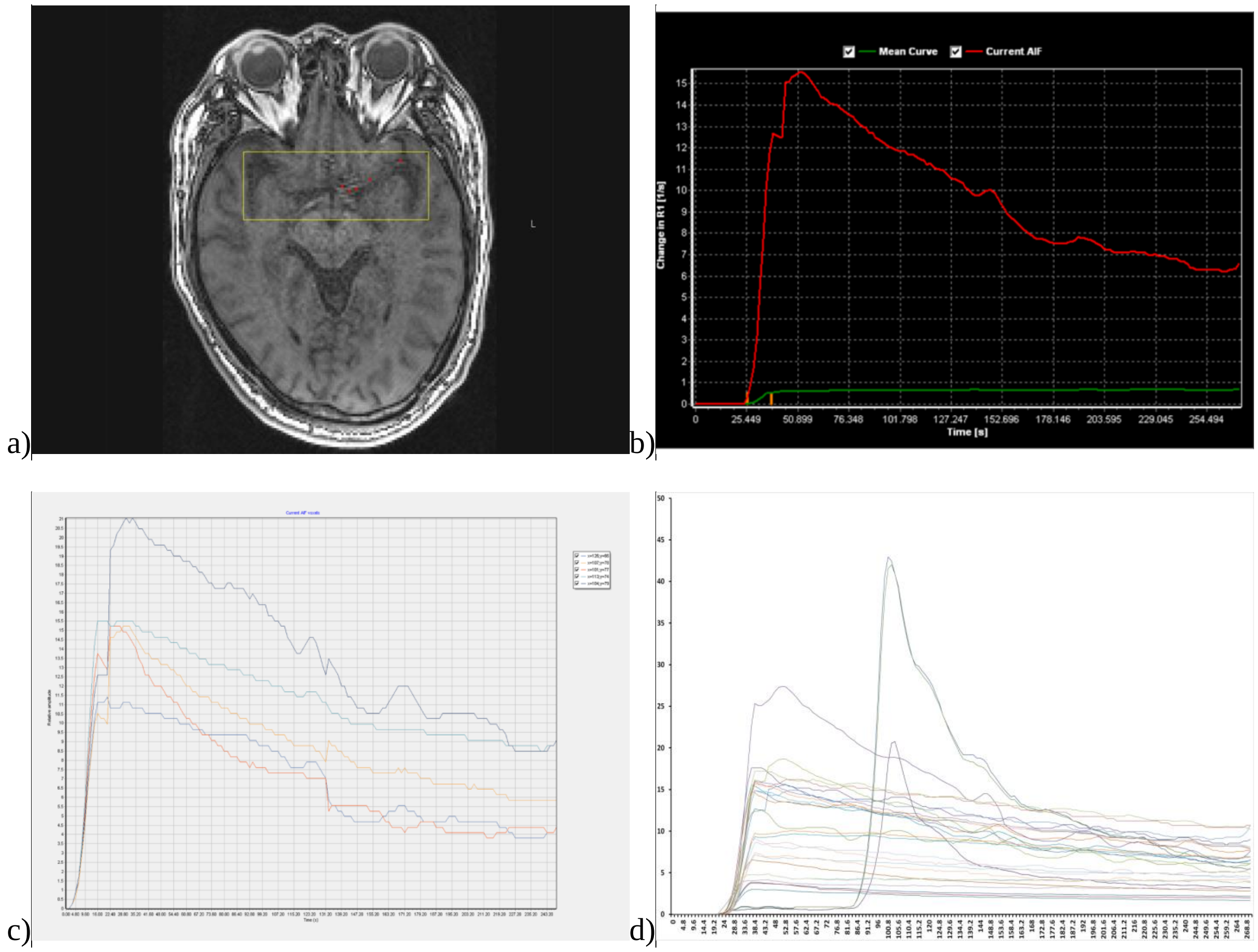

Supplement: Supplementary file 1 — Supplementary Information 1. [file 41598_2021_2450_MOESM1_ESM.pdf]
